# Risk Factors for Mortality in Stroke Patients Admitted to Critical Care Units: A Systematic Review and Meta‐Analysis

**DOI:** 10.1002/brb3.71082

**Published:** 2025-11-29

**Authors:** Lanzhen Chen, Obai Yousef, Amr Elrosasy, Khalid Sarhan, Moaz Elsayed Abouelmagd, Munzer Naima, Alshaimaa Galal, Linda Alkassas, Ahmed A. Abo Elnaga, Ibrahim Serag

**Affiliations:** ^1^ Department of Nursing The Second Affiliated Hospital of Xiamen Medical College Xiamen China; ^2^ Faculty of Medicine Tartous University Tartous Syria; ^3^ Faculty of Medicine Cairo University Cairo Egypt; ^4^ Faculty of Medicine Mansoura University Mansoura Egypt; ^5^ Faculty of Medicine University of Aleppo Aleppo Syria; ^6^ Faculty of Medicine Fayoum University Fayoum Egypt

## Abstract

**Background:**

Stroke is a leading cause of morbidity and mortality worldwide, and patients admitted to intensive care units (ICUs) who require mechanical ventilation face an even higher risk of adverse outcomes, including increased mortality. Effective management in ICUs is crucial to improve patient outcomes.

**Aim:**

This systematic review and meta‐analysis aimed to synthesize existing evidence on the risk factors associated with mortality in stroke patients admitted to any form of critical care units.

**Methods:**

A comprehensive search was conducted across four databases: PubMed, Web of Science, Scopus, and Embase, up to July 9, 2024. Studies were included if they evaluated mortality risk factors in adult stroke patients admitted to critical care units. Data were extracted and analyzed using a random‐effects model to account for heterogeneity. Odds ratios (OR) and 95% confidence intervals (CI) were calculated for various risk factors.

**Results:**

Eighteen studies involving 20,442 patients were included in the meta‐analysis. Age was significantly associated with increased mortality (OR = 1.02; 95% CI: 1.01, 1.04). Lower Glasgow coma scale (GCS) scores were linked to higher mortality (OR = 0.93; 95% CI: 0.86, 1.01), though not statistically significant (*P* = 0.08). Higher National Institutes of Health Stroke Scale (NIHSS) scores showed a significant association with increased mortality (OR = 1.06; 95% CI: 1.03, 1.09), (*p* < 0.0001). Mechanical ventilation and higher body temperature (≥37.5°C) were associated with a higher risk of death (OR = 1.9; 95% CI: 1.65, 2.18) and (OR = 2.03; 95% CI: 1.56, 2.66) respectively. Atrial fibrillation (OR = 1.19; 95% CI: 1.06, 1.34) significantly contributed to mortality risk, while a higher body mass index (BMI) was not associated with a reduced risk of mortality (OR = 0.97; 95% CI: 0.92, 1.02).

**Conclusion:**

This study highlights the critical importance of early identification and targeted management of high‐risk stroke patients in critical care settings. Age, neurological status, respiratory support needs, and specific comorbidities are key factors that clinicians should consider improving survival outcomes. Further research is needed to refine these findings and optimize care strategies for critically ill stroke patients.

## Graphical abstract

Critical risk factors—age, stroke severity, mechanical ventilation, fever, and atrial fibrillation—significantly increase mortality in critical care‐admitted stroke patients, while higher BMI may be protective, emphasizing the need for early risk stratification and targeted management to improve survival outcomes.

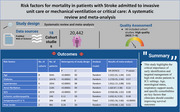

## 1 Introduction

Stroke remains a leading cause of morbidity and mortality worldwide, representing a significant burden on healthcare systems globally (eClinicalMedicine 2023). It is considered the second leading cause of death globally (Hilkens et al. 2024). According to the 2019 global burden of diseases (GBD) there were 12.2 million incident cases and 6.55 million deaths from stroke (Feigin et al. 2021). Stroke patients who are admitted to the intensive care units (ICU) and require mechanical ventilation are at higher risk of adverse outcomes, including high mortality rate (Zhang et al. 2024).

The management of stoke patients in ICUs and critical care units require a multidisciplinary approach to address the acute neurological insult and the associated systemic complications particularly in patients on mechanical ventilation. Those patients are more vulnerable to a wide range of serious complications including respiratory failure, pressure ulcer, aspiration pneumonia, and multi‐organ failure, which can significantly impact their prognosis (Tang et al. 2024). Those patients follow a particular survival pattern with a higher short‐term mortality particularly within the first 30 days. However, if they are managed correctly and survive this critical period, they tend to have relatively better long‐term survival outcomes (Van Valburg et al. 2024).

Several studies have explored predictors that contribute to mortality in stroke patients admitted to ICU (Zhang et al. 2024, Tang et al. 2024, Van Valburg et al. 2024, Fang et al. 2024). Key risk factors include advanced age, where elderly patients are often more susceptible to major complications like pneumonia and multiorgan failure. Chronic disease including hypertension, diabetes and cardiovascular diseases which are more evident in elderly population further exacerbates the risk of mortality in stroke patients (Van Valburg et al. 2024, Shah et al. 2023, Schielke et al. 2005)Additionally, patients with severe respiratory or neurological compromise that require mechanical ventilation are often associated with worse outcomes (Jin et al. 2023, Jeng et al. 2008). Stroke severity, which is typically measured by the National Institutes of Health Stroke Scale (NIHSS) or the Glascow Coma Scale (GCS) is another clinical parameter that is often used to predict the risk of mortality in ICU patients (Wang et al. 2022, Ho et al. 2016). Studies have also shown that patients with a prior history of stoke are at increased risk of mortality when they experience a subsequent stroke (Van Valburg et al. 2024, Jeng et al. 2008).

Understanding those predictors and risk factors in this vulnerable population is essential for optimizing management strategies thereby improving stroke patient outcomes. Therefore, we conducted this systematic review and meta‐analysis aiming to synthesize the available evidence on the risk factors associated with mortality in patients with stroke admitted to any form of critical care units

## 2 Methods

We performed this systematic review and meta‐analysis in accordance with the established guidelines outlined by PRISMA (preferred reporting items for systematic reviews and meta‐analyses) (Page et al. 2021). All authors worked independently on the study retrieval, eligibility evaluation, data extraction, and quality assessment.

### 2.1 Literature Search

We thoroughly searched four databases: PubMed, Web of Science, Scopus, and Embase, until ninth of July 2024 for eligible studies. The search strategy was carried out utilizing medical subject headings (MeSH) and their equivalents. The search terms used were stroke, Intensive care units, critical care, Mechanical ventialt*, extubation, Endotracheal intubation, outcome, risk* mortality, death, surviv* predict, prognos*. The vocabulary and syntax were modified in accordance with the needs of each database, and the Boolean operators “AND” and “OR” were employed to join the terms. The detailed search strategy for the four databases is shown in Supporting Information.

### 2.2 Study Selection

**FIGURE 1:**
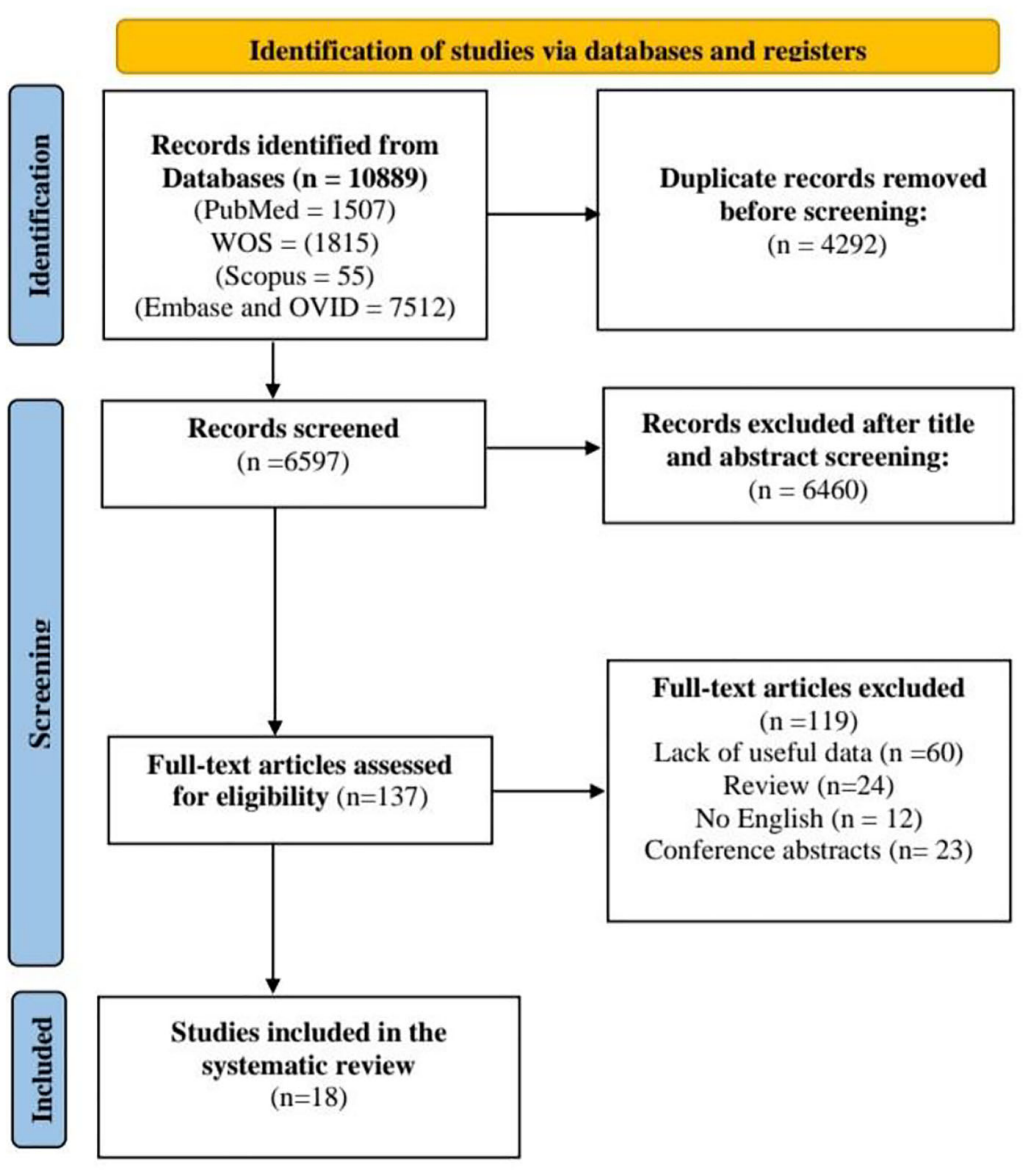
PRISMA flow diagram of articles chosen for the study.

After removing duplicates using Endnote X9, two researchers independently reviewed each study's title and abstract to exclude any irrelevant papers based on our eligibility criteria. Then the full text of studies that met our predetermined inclusion criteria were screened. Any disagreements were settled by discussion and agreement with a third reviewer. A PRISMA flow diagram of the included studies is shown in Figure 1.

### 2.3 Eligibility Criteria

We included clinical studies that only met the following criteria: (1) Any cohort studies (prospective or retrospective), case control, (3) studies measuring risk of mortality in strokepatients admitted to any form of critical care (intensive care unitor invasive unit care, or those receiving mechanical ventilation). Records were excluded if they were: (1) non‐English; (2) other study designs like case reports, case series, nonmedical papers, reviews, conference abstracts, animal or in vitro studies, and pre‐clinical studies; (3) studies with mortality due to heat stroke or electrical shock.

### 2.4 Data Extraction

The following data were extracted from each included study using Excel sheet: Study ID, study design, period, country, follow‐up, measures of associations, statistically significant variables, sample size, rate of mortality and characters of patients (age, number of male and type of stroke, duration of mechanical ventilation and length of ICU

For data extraction related to outcomes, we recorded the odds ratio (OR), relative risk (RR) or hazard ratio (HR) with 95% confidence intervals (Cl) for each variable, including those reported as statistically significant in at least one study. If a multivariate analysis was not available, we extracted data from univariate analysis. In addition, if the study included multiple times, we extracted the outcomes from the last follow‐up.

### 2.5 Risk of Bias Assessment

Two independent researchers used the Newcastle‐Ottawa Quality Assessment Scale (NOS) to evaluate the quality of the included cohort studies, where three broad parameters are used to evaluate each study: selection and representativeness of the study groups; comparability of the groups; and the ascertainment of either the exposure or outcome of interest (Stang, 2010). Each study quality was categorized into one of three groups according to its scores following ranges: 0 to 3, 4 to 6, and 7 to 9. These ranges corresponded to low, medium, and high study quality, respectively.

### 2.6 Data Analysis

If multiple studies are found reporting risk ratios (RR) or hazard ratio (HR) instead of OR, they were converted to OR using the methods described by VanderWeele 2020. The meta‐analysis was conducted using pooled OR with 95% confidence intervals (Cls). We used random‐effects model due to the expected heterogeneity between studies. Higgins *I* ^2^ test was used to measure heterogeneity, where a range of 0–40% was deemed insignificant, 30–60% considered moderate, 50–90% represented substantial, and 75–100% represented considerable heterogeneity. Sensitivity analyses were employed to evaluate the result's robustness and to assess the contribution of each study by excluding studies each at a time. We conducted our statistical analysis using Review Manager V.5.3.

### 2.7 Outcome

We included the variables which were only significant at least in one study and included in two studies at least. This included age, diabetes, hypertension, atrial fibrillation, GCS, Ischemic cardiomyopathy, temperature ≥37.5°C, prior stroke, mechanical ventilation, NIHSS score, BMI, glucose >10, APACHE II, Infarction of the complete MCA territory

## 3 Results

### 3.1 Literature Search

Our search yielded 10889 records. After removing duplicates and screening titles and abstracts, 185 remained for full‐text review. Following a detailed review of the full text, 18 studies, which met the inclusion criteria, were included in our systematic review. The PRISMA flow diagram in Figure 1 illustrates the process of study selection

### 3.2 Characteristics of the Included Studies and Quality Assessment

This meta‐analysis included 18 studies (Van Valburg et al. 2024, Shah et al. 2023, Schielke et al. 2005, Jin et al. 2023, Jeng et al. 2008, Wang et al. 2022, Ho et al. 2016, Furlan et al. 2020, Rordorf et al. 2000, He et al. 2023, Lan et al. 2006, Wang et al. 2018, Santoli et al. 2001, Popat et al. 2018, Viderman et al. 2020, Van Valburg et al. 2018, Navarrete‐Navarro et al. 2003, Berrouschot et al. 2000) investigating risk factors for mortality in stroke patients who received mechanical ventilation (MV), Intensive care unit (ICU), critical care (CC). These studies included a total of 20,442 patients. Most studies measured mortality at various time points, ranging from in‐hospital mortality to 12 months expect one study by Van Valburg 2018 (Van Valburg et al. 2018) investigated mortality over a longer timeframe, with a follow‐up period of approximately 4 years. The reported mortality rate varied from 10% to 81% and it was over in patients required MV. The types of studies included in the analysis were retrospective and prospective studies. However, the characteristics of the included studies and summary of significant variables are described in Table 1. The demographic of the stroke patients in the included studies are presented in Table 2. The included studies were all considered high quality. The total score showed in Table 2, and for more details in Table S1.

**TABLE 1:** The characteristics of the included studies.

Study ID	Study design, period	Country	Follow‐up	Measures of associations	Statistically significant variables
Popat 2018 (Popat et al. 2018)	Retrospective, 2010‐2015	USA	hospital mortality	OR	Age, Volume of Stroke, Altered level of consciousness
Santoli 2001 (Santoli et al. 2001)	Prospective, 1991–1996	France	12 months	RR	Isheamic cardiopathy, coma, bilateral absence of pupillary light reflex, Bilateral absence of corneal reflex, absent fronto‐orbicular reflex.
Schielke 2004 (Schielke et al. 2005)	Retrospective, 1996–1999	Germany	2 months	RR	Age >60 years, myocardial infarction, GCS < 10 on admission, complete MCA infarction, Isheamic cardiopathy
Berrouschot 2000 (Berrouschot et al. 2000)	Prospective, 1994–1997	Germany	3 months	OR	—
Rordorf 2000 (Rordorf et al. 2000)	Retrospective, 1992–1995	USA	hospital mortality	OR	APACHE II, GCS, temperature, WBC, arterial PH
Jeng 2008 (Jeng et al. 2008)	Prospective, 2002‐2006	Taiwan	3 months	HR	IS: NIHSS, requiring ventilator aid, Hematocritb30%. ICH: NIHSS, requiring ventilator aid, ICH volume ≥30 mL
Furlan, 2020 (Furlan et al. 2020)	Prospective 2012 ‐2016	Baraziel	1 week	OR	Statins
Jin 2023 (Jin et al. 2023)	Retrospective, 2008‐2019	China	12 months	OR	Age, marital status (widowed, other), dementia, malignant cancer, metastatic solid tumor, heart rate, respiratory rate, oxygen saturation, WBC, anion gap, mannitol injection, invasive mechanical ventilation, first day GCS
Wang, 2018 (Wang et al. 2014)	Retrospective, 2012–2013	China	Hospital mortality	OR	Age, gastrointestinal hemorrhage, AKI satage 1,2,3
Lan, 2006 (Lan et al. 2006)	Prospective 2000–2002	Taiwan	ICU mortality	OR	Reaction level scale score, modified acute physiology score.
Wang, 2022 (Wang et al. 2022)	Retrospective,2013–2007	China	6 months	OR	APACHE II score, GCS score, protein provision
Van valburg, 2024 (Van Valburg et al. 2024)	Retrospective,2010–2019	Netherlands	1 months	OR	IS: Age, NYHA class IV, APACHE to III APS, GCS score, mechanical ventilation, AKF. ICH: Age, APACHE to III APS, GCS score, specialized neurosurgical center, Malignanciesa.
Handschu 2005 (Handschu et al. 2005)	Prospective, 1 year	Germany	12 months	HR	SAPS score, SAPS (II) score, GCS score
Ho 2016 (Ho et al. 2016)	Retrospective, 2009–2011	Taiwan	hospital mortality	OR	IS: Age, NIHSS, systolic BP, WBC. HS: NIHSS, systolic BP. heart disease history.
Navarro 2003 (Navarrete‐Navarro et al. 2003)	Prospective 1999–1999 (6 months)	Spain	12 months	OR	AGE, APACHE III
Viderman 2020 (Viderman et al. 2020)	Retrospective, 2009–2013	Kazakhstan	hospital mortality	HR	Male, cerebral edema
van Valburg 2018 (Van Valburg et al. 2018)	Retrospective, 2010–2012	United Kingdom	48 months	HR	Age (50–64, 65–79, ref <50), acute physiology and chronic health evaluation 2 score, reason for admission: impaired consciousness (GCS ≤8) (reference = neuromonitoring), atrial fibrillation, previous cerebrovascular accident, temperature, GCS sum score at admission, GCS sum score after 24 h, difference GCS day 1—GCS admission (reference = no difference), decreased GCS after 24 h, brainstem reflexes (reference = presence), brainstem involvement (reference = no)
Panda 2023 (Shah et al. 2023)	Prospective, 2021–2023	India	1 months	OR	IS: Atrial fibrillation, diabetes, hypertension, CAD/MI, previous stroke/TIA, delayed EMD arrival. ICH: Hypertension, delayed EMD arrival, diabetes, CAD/MI.

MCA: middle cerebral artery, APACHE: acute physiology and chronic health evaluation, GCS: Glasgow coma scale, ICH: intracerebral hemorrhage, IS: ischemic stroke, HS: Hemorrhagic stroke, BMI: Body mass index, NIHSS: National Institute Health Stroke Scale, WBC, white blood cell count., APS, acute physiology score, CAD: coronary artery disease, MI: myocardial infarction, TIA: transient ischemic attack, EMD: emergency medicine department, AKF: acute renal failure, AKI: BP: blood pressure, acute kidney injury. OR: odds ratio, RR: relative risk, HR: hazard ratio.

**TABLE 2:** The characteristics of patients and included studies and the total NOS.

Study ID	Population	Sample size	Mortality rate	Age years (SD)	Mean duration of mechanical ventilation—days (SD)	Mean duration of length of ICU—days (SD)	Male *N* (%)	Total NOS
Popat 2018 (Popat et al. 2018)	All types	226	56.60%	60.3 (14.3)	6.5 (5.9)	NR	119 (52.7)	7
Santoli 2001 (Santoli et al. 2001)	Ishamic	58	72.40%	65 (13)	8.6 (8.8)	NR	34 (59)	8
Schielke 2004 (Schielke et al. 2005)	Ishamic	101	44%	63.8 (12.4)	NR	NR	56 (55.4)	7
Berrouschot 2000 (Berrouschot et al. 2000)	Ischemic	42	81%	62 (12)	7.16 (7.58)	NR	27 (64)	8
Rordorf 2000 (Rordorf et al. 2000)	Ischemic	63	20.60%	64.36 (13.07)	NR	4	31 (49.2)	7
Jeng 2008 (Jeng et al. 2008)	All types,	850	17%	65.3 (14.4)	NR	9.4 (8.8)	491 (58)	8
Furlan, 2020 (Furlan et al. 2020)	Ischemic	97	24%	67.37 (97)	NR	NA	54 (55.6)	7
Jin 2023 (Jin et al. 2023)	Ischemic	1443	18.09%	68.26 (15.63)	NR	4.85 (4.37)	761 (52.7)	8
Wang, 2018 (Wang et al. 2018)	All types	647	10.00%	58.1 (13.6)	NR	NR	350 (54.1)	7
Lan, 2006 (Lan et al. 2006)	Ischemic	231	14.70%	68.44 (11.15)	NR	68.44(11.18)	103 (44.6)	7
Wang, 2022 (Wang et al. 2022)	All types	208	13.9%	63.0 (5.25)	NR	12.67 (7.47)	141 (67.8)	8
Van valburg, 2024 (Van Valburg et al. 2024)	All types	14303	32.90%	69 (14.087), 62.33 (16.31)	NR	NR	8136 (56.9)	8
Handschu 2005 (Handschu et al. 2005)	All types	90	67.8%	64.3 (10.4)	7.5 (6.2)	NR	45 (90)	8
Ho 2016 (Ho et al. 2016)	All types	1416	18%	65.75 (15.75)	NR	NR	847 (59.8)	7
Navarro 2003 (Navarrete‐Navarro et al. 2003)	All types	132	53%	55.7 (15.8)	NR	13 (12.5)	81 (61)	8
Viderman 2020 (Viderman et al. 2020)	All types	148	37.20%	63.3 (14.2)	NR	7 (11.167)	66 (44.6)	7
van Valburg 2018 (Van Valburg et al. 2018)	All types	131	58.80%	70 (18)	NR	6(1.3)	76 (58)	8
Panda 2023 (Shah et al. 2023)	All types	256	16%	58.1 (15.69)	NR	NR	198 (77.34)	8

ICU: intensive care units, NR: not report.

### 3.3 Risk Factors for Mortality in Patients With Stroke Admitted to Critical Care

Among the studies included in the meta‐analysis, some reported data for ischemic stroke and hemorrhagic stroke separately. To maintain consistency and clarity, studies that reported separate data for ischemic stroke and hemorrhagic stroke were classified as two distinct studies within the meta‐analysis. Studies reporting on ischemic stroke were designated as Study ID (a), while studies reporting on hemorrhagic stroke were labeled as Study ID (b).

**FIGURE 2:**
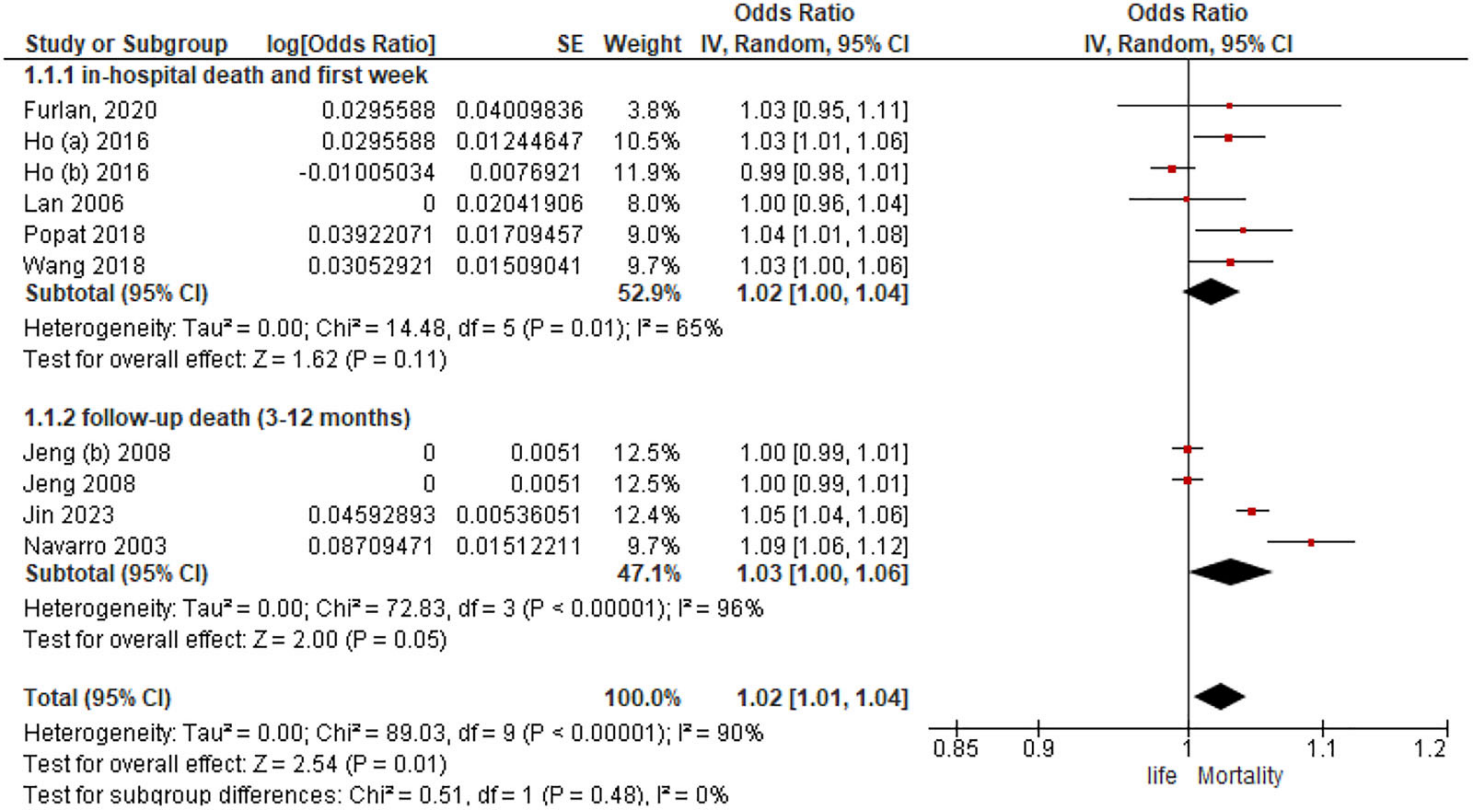
Forest plot analysis of the impact age on mortality.

The mortality risk variables for stroke patients admitted tocritical care units are listed in Table 3 of the meta‐analysis. To explore potential variations in the association between age and mortality across different time points, we conducted a subgroup analysis based on the time of measurements. The meta‐analysis included two subgroup hospital stay up to 7 days and follow‐up 3–12 months. In the subgroup of hospital stay up to 7 days, the analysis of 5 studies showed no significant difference between age and mortality with an OR = 1.02: 95%Cl (1, 1.04), *P* = 0.11. However, the heterogeneity test showed significant heterogeneity (*I* ^2^ = 65%, *P* = 0.01) (Figure 2). To address the observed heterogeneity, we explored potential sources of variation by conducting sensitivity analysis. We found that heterogeneity was driven by Ho 2016 (b). After exclusion of this study, the associations between age and mortality became statistically significant OR = 1.03; 95%Cl (1.01, 1.04), *P* = 0.0003 with no heterogeneity (*I* ^2^ = 0%, *P* = 0.67) (Figure S1). In the subgroup of follow‐up (3–12 months), the analysis of 3 studies showed borderline statistical significance for association between age and mortality with an OR = 1.03; 95%Cl (1, 1.06), *P* = 0.05. However, the heterogeneity test showed significant heterogeneity (*I* ^2^ = 96%, *P* < 0.00001) (Figure 2). We found that heterogeneity was driven by Jin 2023 and Navaro 2023. Upon excluded these studies, the associations between age and mortality are still not statistically significant OR = 1; 95%Cl (0.99, 1.01), *P* = 1 with no heterogeneity (*I* ^2^ = 0%, *P* = 1) (Figure S1). Overall, the analysis showed a significant association between age and mortality with OR = 1.02; 95%Cl (1.01, 1.04), *P* = 0.01 (Figure 2), and showed borderline statistically significant difference after solving heterogeneity OR = 1.01; 95%Cl (1, 1.02), *P* = 0.05 (Figure S1).

**TABLE 3:** Meta‐analysis for risk factors of mortality in stroke patients who required critical care.

Risk factors	No. of cohorts	No. of patients	Heterogeneity of study design		Analysis model	Results of meta‐analysis	
			*P*	*I* ^2^		OR (95%Cl)	*P*‐values
Age	8	5041	<0.00001	90	Random	1.02 (1.01, 1.04)	0.01
Diabetes	7	10,476	<0.00001	99	Random	1.27 (0.55, 2.94)	0.57
hypertension	6	2054	<0.00001	98	Random	1.81 (1.13, 2.88)	0.01
Atrial fibrillation	7	2185	<0.00001	99	Random	1.6 (0.87, 3.38)	0.22
GCS	6	2870	<0.00001	97	Random	0.93 (0.86, 1.01)	0.08
Ischemic cardiomyopathy	2	249	0.12	59	Random	1.63 (1.8, 3.31)	0.18
Temperature ≥37.5°C	2	394	0.48	0	Fixed	2.03 (1.56, 2.66)	<0.00001
Prior stroke	2	981	0.24	30	Random	1.07 (0.88, 1.26)	0.52
Mechanical ventilation	3	16,596	0.002	76	Random	1.98 (1.47, 2,67)	<0.00001
NIHSS score	4	3010	<0.00001	86	Random	1.06 (1.03, 1.09)	<0.00001
BMI	4	1315	0.0003	79	Random	0.97 (0.92, 1.02)	0.24
Glucose >10	2	902	0.57	0	Fixed	1.26 (0.99, 1.61)	0.06
APACHE II	2	8554	0.0006	91	Random	1.2 (0.89,1.62)	0.24
Infarction of the complete MCA territory	2	153	0.55	0	Fixed	2.41 (1.32, 4.42)	0.004

MCA: middle cerebral artery, APACHE: acute physiology and chronic health evaluation, GCS: Glasgow coma scale, BMI: body mass index, NIHSS: National Institute Health stroke scale.

A significant heterogeneity was observed among studies in diabetes, hypertension, atrial fibrillation, GCS, mechanical ventilation, NIHSS score, BMI, APACHE II as shown in Table 3 which also offers values of OR and 95% Cl (Figures S2, S4, S6, S8, S9, S11, S12, and S19). After removing panda 2023 study, the heterogeneity was solved among studies in diabetes, hypertension, atrial fibrillation. Pooled analysis of studies indicated that diabetes has no statically significant association with mortality OR = 1.27; 95%Cl (0.55, 2.94), *P* = 0.57, and still insignificant after sensitivity analysis OR = 1.13; 95%Cl (0.97, 1.32), *P* = 0.11 (Figures S2 and S3). Hypertension was not significant OR = 1.07; 95%Cl (0.87, 1.3), *P* = 0.54 (Figure S5). Regarding atrial fibrillation, pooled analysis of 8 studies revealed non‐significant association with mortality outcomes with substantial heterogeneity OR = 1.06; 95%Cl (0.76, 3.38), (*P* = 0.22). Then, it showed dramatical shift to statistical significance after exclusion of Panda 2023, OR = 1.19; 95%Cl (1.06, 1.34), (*P* = 0.003) (Figures S6 and S7). For mechanical ventilation (MV), the heterogeneity was moderate (*I* ^2^ = 55%) and completely resolved after removing Van Valburg 2024. MV was significantly associated with an increased risk of mortality OR = 1.98; 95%Cl (1.47, 2.67), *P* < 0.00001 (Figures S9 and S10), while BMI was associated with a decreased risk OR = 0.95; 95%Cl (0.92, 0.98), *P* = 0.002 after removal of Jeng 2008 (Figures S11 and S12). The analysis showed that NIHSS was significantly associated with mortality with OR = 1.06; 95%Cl (1.03, 1.09), *P* < 0.00001 with evidence of heterogeneity (*I* ^2^ = 86%, *P* < 0.00001) (Figure S13), and GCS score was not statically significant OR = 0.93; 95%Cl (0.86, 1.01), *P* = 0.08 with heterogeneity (*I* ^2^ = 97%, *P* < 0.00001) (Figure S8). The source for heterogeneity in GCS score, and NIHSS score was not addressed. Notably, ischemic cardiomyopathy, and history of prior stroke showed statical insignificance for association with mortality OR = 1.63; 95%Cl (0.8, 3.31), *P* = 0.18 and OR = 1.07; 95%Cl (0.88, 1.29), *P* = 0.52 respectively (Figures S14 and S17). Similarly, glucose >10 and APACHE II were not significant predictors (Figures S18 and S19). On the other hand, infarction of the complete MCA territory and temperature ≥37.5°C had statistically significant association with mortality OR = 2.41; 95%Cl (1.32, 4.42), *P* = 0.004, and OR = 2.03; 95%Cl (1.56, 2.66), *P* < 0.00001 respectively (Figures S15 and S16). All values of OR and 95 %Cl showed in Table 3.

## 4 Discussion

The results of this systematic review and meta‐analysis highlight several key risk factors for mortality in stroke patients admitted to any form of critical care. The analysis, which included 18 studies with a total of 20,442 patients, revealed that mortality rates varied widely, with higher rates observed among patients on mechanical ventilation, suggests that the severity of the patient's condition plays a significant role in their outcomes. Age emerged as a significant risk factor, particularly in the short‐term hospital stay period, indicating that older patients are at a higher risk of mortality soon after stroke when critical care is required. The analysis also identified atrial fibrillation as an important predictor of mortality, suggesting that these underlying conditions may complicate recovery and contribute to poorer outcomes. Additionally, higher scores on the NIH Stroke Scale, which indicates more severe neurological impairment, were associated with increased mortality, emphasizing the impact of stroke severity on patient survival. Mechanical ventilation, often a marker of severe respiratory or neurological compromise, was linked to a greater risk of death, reinforcing the idea that patients requiring this level of support are in a more precarious state. Additionally, a higher body mass index appeared to be protective, potentially reflecting the role of proper nutrition in hospitals and ICUs and overall health status in enhanced recovery from stroke. Other factors, such as, complete MCA territory infarction, and elevated body temperature, were also associated with increased mortality, highlighting the importance of comprehensive monitoring and management of these conditions in critically ill stroke patients. Additionally, ischemic cardiomyopathy showed higher risk for mortality, however, it did not reach statistical significance. On the other hand, factors like hypertension, diabetes, GCS score, prior stroke, glucose levels, and APACHE II score were not found to be significant predictors, suggesting that while these are important clinical considerations, they may not independently influence mortality risk in the same way as the other identified factors.

In comparison with existing literature, our findings align with the results reported by Wang et al. in their nationwide multicenter study on ICU patients with heat stroke, where key physiological parameters such as creatinine (Cr), aspartate aminotransferase (AST), and systolic blood pressure (SBP) were identified as significant predictors of in‐hospital mortality, with odds ratios of 1.005 (95% CI: 1.001–1.008), 1.002 (95% CI: 1.000–1.003), and 0.981 (95% CI: 0.969–0.992), respectively. Similarly, our meta‐analysis identified several clinical parameters, such as higher NIHSS scores, mechanical ventilation, and lower BMI, as significant predictors of mortality in stroke patients, with NIHSS showing a strong association with increased mortality (OR = 1.06; 95% CI: 1.03, 1.09). The Wang et al. study emphasized the importance of renal and hepatic dysfunction as reflected by elevated Cr and AST levels, whereas our analysis highlighted the impact of neurological impairment and respiratory support on mortality risk in stroke patients. Both studies reinforce the concept that specific clinical measures are crucial for predicting outcomes in critically ill patients. However, while Wang et al. focused on biochemical markers relevant to heat stroke, our study emphasized the relevance of neurological and respiratory factors in the context of stroke, indicating that risk factors for mortality are highly dependent on the specific condition being treated (Wang et al. 2023)

Our findings align with those reported by van Valburg et al. in their study on predicting 30‐day mortality in ICU patients with ischemic stroke and ICH. In their study, the 30‐day mortality rate was 27% for ischemic stroke and 41% for ICH, which closely mirrors the mortality rates observed in our meta‐analysis, where mortality rates varied from 10% to 81%, with higher rates typically observed in patients requiring mechanical ventilation. Van Valburg et al. identified several critical predictors of 30‐day mortality, including age, GCS score, and the application of mechanical ventilation. For instance, a low GCS score (3 to 8) was associated with a 14.5‐fold increase in mortality risk in ischemic stroke patients and a 12.6‐fold increase in ICH patients. However, our meta‐analysis found that lower GCS scores were associated with higher mortality but it did not reach statistical significance. Additionally, van Valburg et al. reported that mechanical ventilation was a significant predictor of mortality, with an OR of 1.37 for ischemic stroke and 1.69 for ICH. This finding is consistent with our results, where mechanical ventilation was associated with an increased mortality risk (OR = 1.9; 95% CI: 1.65, 2.18). Both studies also highlighted the significance of age, with van Valburg et al. showing that each increase in age category significantly raised the odds of mortality, like our finding that age was significantly associated with mortality, particularly in short‐term hospital stays. The predictive accuracy of van Valburg et al.'s models was high, with AUCs of 0.85 for both ischemic stroke and ICH, which is comparable to the strong associations and predictive values observed in our analysis for various risk factors. This consistency across studies underscores the reliability of age, and mechanical ventilation as key predictors of mortality in critically ill stroke patients (Van Valburg et al. 2024).

Our findings are consistent with those of Navarrete‐Navarro et al., who reported a 1‐year mortality rate of 53.8% among ICU‐admitted stroke patients, with the highest mortality in ischemic stroke (66%), followed by intracerebral hemorrhage (54%) and subarachnoid hemorrhage (39%). However, our meta‐analysis showed that higher APACHE II scores, and low GCS scores were non‐statistically significant predictors of mortality which may be attributed to limited studies in analysis for APACHE II outcome. Additionally, The study by Navarrete‐Navarro et al. highlights the critical importance of age as predictive factor in predicting long‐term outcomes for stroke patients in ICU settings which is consistent with our findings (Navarrete‐Navarro et al. 2003).

The findings of this meta‐analysis have significant implications for managing stroke patients in critical care. Age is significant factor contributing to mortality from stroke as older patients are at a higher risk of mortality and may benefit from more intensive monitoring and tailored interventions. The strong association between higher temperature with increased mortality underscores the need for early identification of high‐risk patients and prompt the importance of careful monitoring of body temperature in critical care. Moreover, careful management of mechanical ventilation is crucial, with efforts to minimize its duration and consider non‐invasive options when possible. Additionally, comprehensive management of comorbidities like atrial fibrillation is essential to reduce mortality risk, while attention to nutritional status, particularly avoiding significant weight loss, could also improve outcomes.

## 5 Limitations

The limitations of this study should be acknowledged to provide a balanced interpretation of the findings. Firstly, the meta‐analysis included studies with varying methodologies, including differences in patient populations, treatment protocols, and outcome measures, which may introduce heterogeneity and affect the generalizability of the results. Despite efforts to address heterogeneity through sensitivity analyses, some residual variability may remain. Secondly, the studies included were predominantly observational, which can limit the ability to establish causality between identified risk factors and mortality outcomes. Additionally, our analysis was limited to studies published in English which may introduce language bias. Finally, we couldn't perform subgroup analysis based on the type of stroke and critical care admission policies due to lack of individualized data and insufficient data of included studies.

## 6 Conclusion

This systematic review and meta‐analysis identified several key risk factors associated with mortality in stroke patients admitted to critical care units, including age, higher body temperature, the need for mechanical ventilation, and the presence of comorbidities such as atrial fibrillation. These findings underscore the critical importance of early identification and targeted management of high‐risk patients to improve outcomes in this vulnerable population. The study also highlights the role of age as a significant predictor for mortality. Therefore, comprehensive care plans and continuous monitoring for older and frail patients are required for this high‐risk group. While the findings provide valuable insights for clinical practice, the limitations related to study heterogeneity and observational design suggest that further research is necessary to confirm these results and guide more effective interventions in critical care settings for stroke patients.

## Author Contributions

Obai Yousef, Amr Elrosasy, Moaz Elsayed Abouelmagd: conceptualization and methodology. Obai Yousef, Amr Elrosasy, Munzer Naima, Alshaimaa Galal, Linda Alkassas: investigation and data curation. Moaz Elsayed Abouelmagd: formal analysis. Ibrahim Serag, Khalid Sarhan, Obai Yousef and Ahmed A. Abo Elnaga: Writing—Original Draft. Obai Yousef, Amr Elrosasy and Ibrahim Serag: Supervision. Amr Elrossasy, Obai Yousef: Project administration. Ahmed A. Abo Elnaga, Ibrahim Serag, and Lanzhen Chen: Writing—Review & Editing. All authors read and approved the final content.

## Funding

The authors have nothing to report.

## Conflicts of Interest

The authors declare no conflicts of interest.

## Supporting information




**Supplementary information**: brb371082‐sup‐0001‐SuppMat.docx

## Data Availability

All data generated or analyzed during this study are included in this published article.
