# Supplementary material for: Risk Factors for Mortality in Stroke Patients Admitted to Critical Care Units: A Systematic Review and Meta‐Analysis
Source: Brain Behav. 2025 Nov 29;15(12):e71082. doi: 10.1002/brb3.71082 (PMC12664908; doi:10.1002/brb3.71082)
Supplement: Supplementary file 1 — Supplementary information: brb371082‐sup‐0001‐SuppMat.docx [file BRB3-15-e71082-s001.docx]

| Study ID | Type of study | Selection | | | | | Comparability | | Exposure | | | |  | |  |
| --- | --- | --- | --- | --- | --- | --- | --- | --- | --- | --- | --- | --- | --- | --- | --- |
|  |  | Representativeness of the exposed cohort | Selection of the non-exposed cohort | Ascertainment of exposure | Demonstration that outcome of interest was not present at the start of the study | Comparability of cohorts on the basis of the design or analysis | | Assessment of outcome | | Was follow-up long enough for outcomes to occur | Adequacy of follow-up of cohorts | Total score | | Overall quality | |
| Santoli 2001 | Prospective | ⋆ | ⋆ | ⋆ | ⋆ | ⋆ | | ⋆ | | ⋆ | ⋆ | 8 | | Good quality | |
| Schielke 2004 | Retrospective | ⋆ | ⋆ | ⋆ | ⋆ | ⋆ | | ⋆ | | ⋆ | ⋆ | 7 | | Good quality | |
| Berrouschot 2000 | Prospective | ⋆ | ⋆ | ⋆ | ⋆ | ⋆ | | ⋆ | | ⋆ | ⋆ | 8 | | Good quality | |
| Rordorf 2000 | Retrospective | ⋆ | ⋆ | ⋆ | ⋆ | ⋆ | | ⋆ | |  | ⋆ | 7 | | Good quality | |
| Jeng 2008 | Prospective | ⋆ | ⋆ | ⋆ | ⋆ | ⋆ | | ⋆ | | ⋆ | ⋆ | 8 | | Good quality | |
| Furlan, 2020 | Prospective | ⋆ | ⋆ | ⋆ | ⋆ | ⋆ | | ⋆ | |  | ⋆ | 7 | | Good quality | |
| Jin 2023 | Retrospective | ⋆ | ⋆ | ⋆ | ⋆ | ⋆ | | ⋆ | | ⋆ | ⋆ | 8 | | Good quality | |
| Wang, 2018 | Retrospective | ⋆ | ⋆ | ⋆ | ⋆ | ⋆ | | ⋆ | |  | ⋆ | 7 | | Good quality | |
| Lan, 2006 | Prospective | ⋆ | ⋆ | ⋆ | ⋆ | ⋆ | | ⋆ | |  | ⋆ | 7 | | Good quality | |
| Wang, 2022 | Retrospective | ⋆ | ⋆ | ⋆ | ⋆ | ⋆ | | ⋆ | | ⋆ | ⋆ | 8 | | Good quality | |
| Van valburg, 2024 | Retrospective | ⋆ | ⋆ | ⋆ | ⋆ | ⋆ | | ⋆ | | ⋆ | ⋆ | 8 | | Good quality | |
| Handschu 2005 | Prospective | ⋆ | ⋆ | ⋆ | ⋆ | ⋆ | | ⋆ | | ⋆ | ⋆ | 8 | | Good quality | |
| Ho 016 | Retrospective | ⋆ | ⋆ | ⋆ | ⋆ | 3 | | ⋆ | |  | ⋆ | 7 | | Good quality | |
| Navarro 2003 | Prospective | ⋆ | ⋆ | ⋆ | ⋆ | ⋆ | | ⋆ | | ⋆ | ⋆ | 8 | | Good quality | |
| Viderman 2020 | Retrospective | ⋆ | ⋆ | ⋆ | ⋆ | ⋆ | | ⋆ | |  | ⋆ | 7 | | Good quality | |
| Van valburg 2018 | Retrospective | ⋆ | ⋆ | ⋆ | ⋆ | ⋆ | | ⋆ | | ⋆ | ⋆ | 8 | | Good quality | |
| Panda 2023 | Prospective | ⋆ | ⋆ | ⋆ | ⋆ | ⋆ | | ⋆ | | ⋆ | ⋆ | 8 | | Good quality | |

**Table 1.** Methodological quality assessment of the included 8 cohort studies, based on the NOS for assessing the quality of observational studies


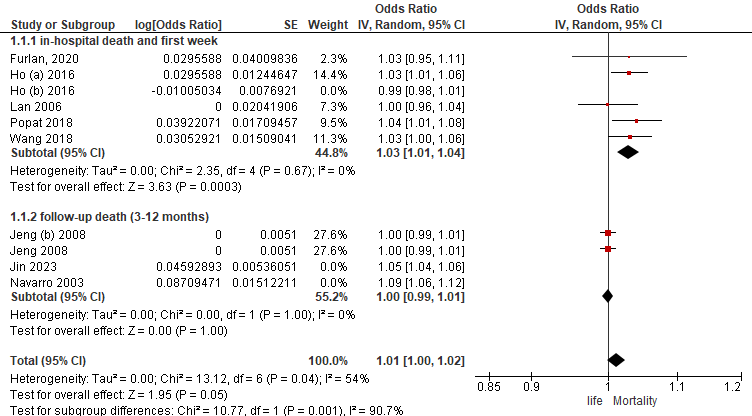


**Figure.1** forest plot of the impact Age on mortality after sensitivity analysis excluding navaro 2003, Jin 2023, HO (b) 2016.


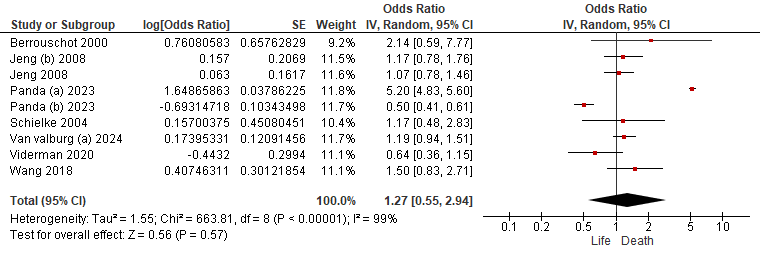


**Figure.2**. Forest plot analysis of the impact diabetes on mortality.


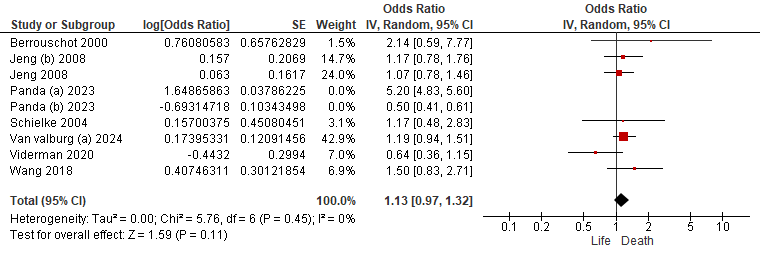


**Figure.3**. Forest plot analysis of the impact diabetes on mortality after sensitivity analysis excluding panda 2023

**
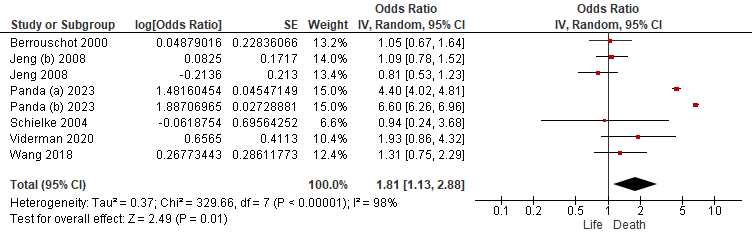
 Figure.4**. Forest plot analysis of the impact hypertension on mortality

**
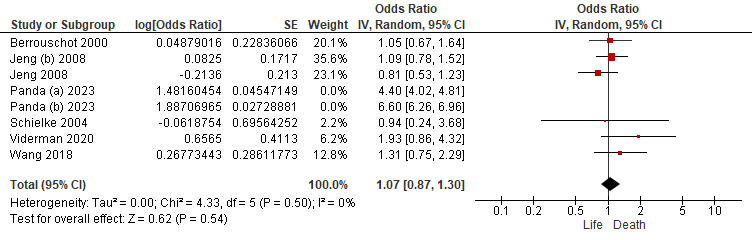
 Figure.5.** Forest plot analysis of the impact hypertension on mortality after sensitivity analysis excluding panda 2023


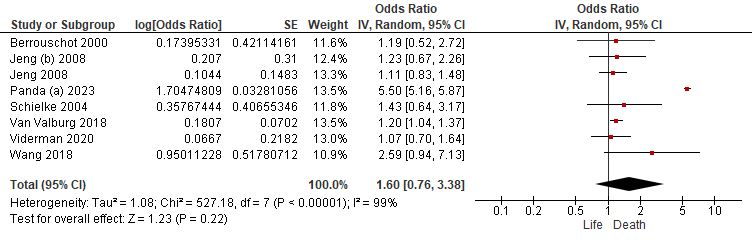


**Figure.6**. Forest plot analysis of the impact atrial fibrillation on mortality


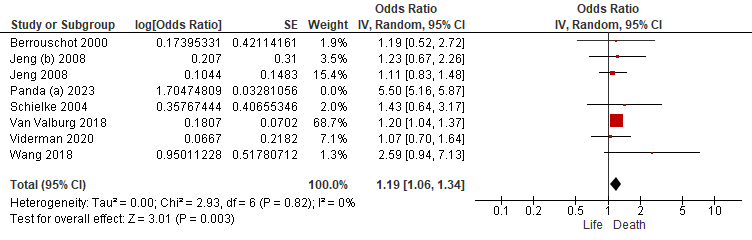


**Figure.7**. Forest plot analysis of the impact atrial fibrillation on mortality after sensitivity analysis excluding panda 2023


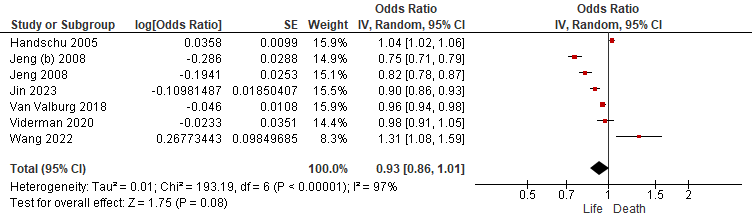


**Figure.8**. Forest plot analysis of the impact GCS score on mortality.


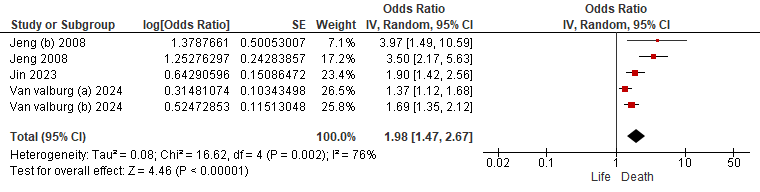
 **Figure.9.** Forest plot analysis of the impact Mechanical ventilation on mortality.


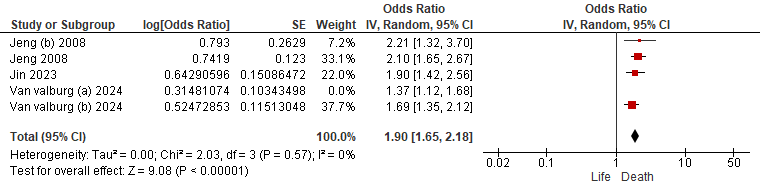


**Figure.10**. Forest plot analysis of the impact mechanical ventilation on mortality after sensitivity analysis excluding Van Valburg 2024.

**
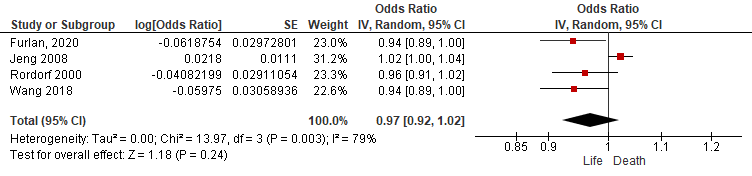
 Figure.11.** Forest plot analysis of the impact BMI on mortality


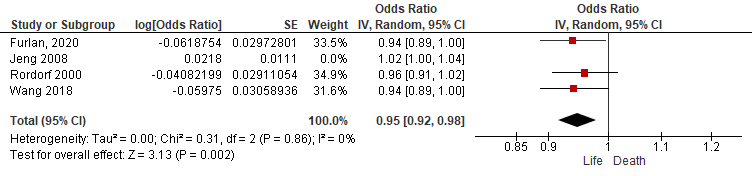


**Figure.12**. Forest plot analysis of the impact BMI on mortality after sensitivity analysis excluding Jeng 2008.

**
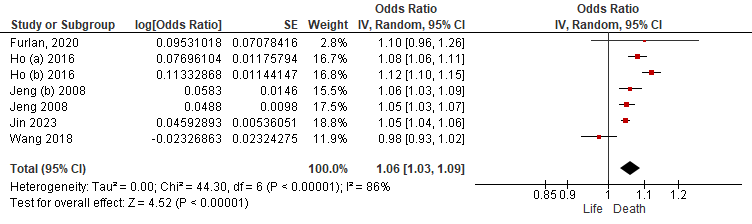
Figure.13.** Forest plot analysis of the impact NIHSS score on mortality.


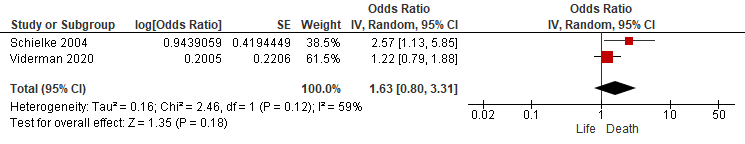


**Figure.14.** Forest plot analysis of the impact Ischemic cardiomyopathy on mortality.


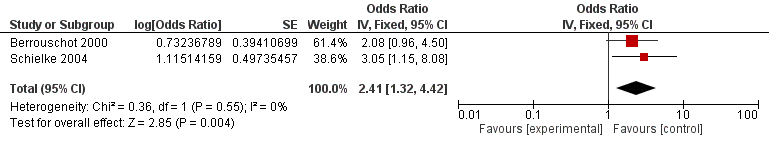


**Figure.15.** Forest plot analysis of the impact Infarction of the complete MCA territory on mortality.


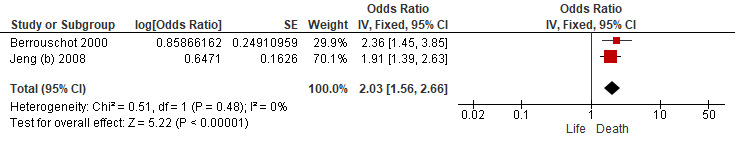
 **Figure.16.** Forest plot analysis of the impact temperature≥37.5 °C on mortality.


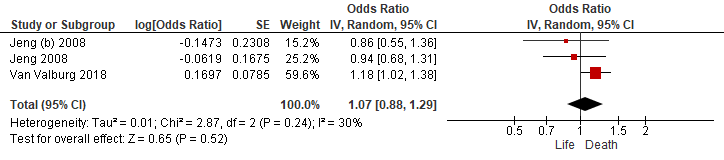
 **Figure.17.** Forest plot analysis of the impact Prior stroke on mortality.

**
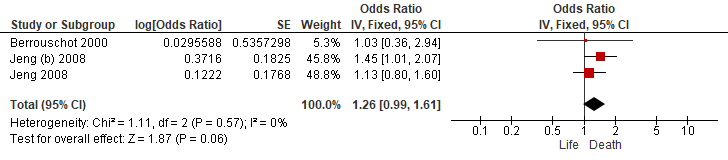
 Figure.18.** Forest plot analysis of the impact glucose >10 on mortality


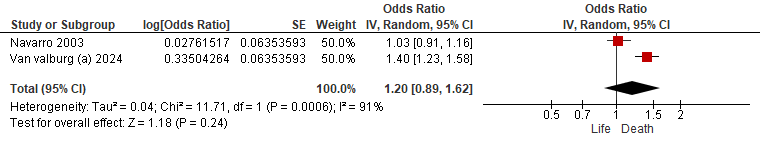


**Figure.19.** Forest plot analysis of the impact APACHE II on mortality
